# Efficacy analysis of electroacupuncture plus TDP in the treatment of peripheral facial paralysis: a systematic review and meta-analysis

**DOI:** 10.3389/fneur.2024.1450312

**Published:** 2024-11-27

**Authors:** Hua-Wei Gao, Qing-Chang Xia, Zhi-Hong Li, Wei Chen, Yan Lu

**Affiliations:** ^1^Department of Acupuncture Moxibustion Massage Rehabilitation and Healthcare, Shandong College of Traditional Chinese Medicine, Yantai, Shandong, China; ^2^College of Acupuncture Moxibustion and Massage, Shandong University of Traditional Chinese Medicine, Jinan, Shandong, China; ^3^Department of Neurology, Shijingshan Teaching Hospital of Capital Medical University, Beijing Shijingshan Hospital, Beijing, China

**Keywords:** peripheral facial paralysis, electroacupuncture, TDP, meta-analysis, complementary therapies

## Abstract

**Objective:**

This study intends to carry out a systematic review and meta-analysis of electroacupuncture combined with TDP in the treatment of peripheral facial paralysis.

**Methods:**

CNKI, VIP, Wanfang, PubMed, Embase and Cochrane databases were searched for literatures on randomized or quasi-randomized controlled trials of electroacupuncture combined with TDP in the treatment of peripheral facial paralysis, and the references of the included studies were searched. Meta-analysis was performed using Stata15.0 software after risk of bias, quality assessment, and data extraction of the included articles by two reviewers independently.

**Results:**

Fifteen articles were finally included, with approximately 1,568 participants (920 in the treatment group and 648 in the control group). Meta-analysis showed that the effective rate of electroacupuncture combined with TDP in the treatment of peripheral facial paralysis was not significantly different from other treatment methods ([*RR* = 1.05, 95%*CI* (0.97, 1.12), *p* = 0.226]), and the recovery rate was better than other treatment methods ([*RR* = 1.14, 95%*CI* (1.05, 1.24), *p* = 0.002]). Subgroup analysis showed that when stratified by the inclusion of minors in the study population, it was observed that in studies including minors, the combination of electroacupuncture and TDP therapy demonstrated superior efficacy in treating peripheral facial paralysis compared to other therapeutic modalities [OR = 1.14, 95% CI (1.03, 1.25), *p* = 0.011]. Conversely, in studies where the population comprised solely adults, no significant difference was found between the combination therapy and other treatments [OR = 1.15, 95% CI (0.99, 1.33), *p* = 0.059]; whether electroacupuncture alone or other treatment methods, the recovery rate of electroacupuncture combined with TDP in the treatment of peripheral facial paralysis was better than other methods.

**Conclusion:**

Electroacupuncture combined with TDP is superior to other treatment methods in the treatment of peripheral facial paralysis.

## 1 Introduction

Peripheral facial paralysis, also known as idiopathic facial nerve palsy and Bell’s palsy, represents a prevalent neuropathy of the facial nerve (1). Clinically, it primarily manifests as impaired motor function of the facial muscles on the affected side, with incomplete or complete paralysis of the expression muscles on the affected side (2). Peripheral facial paralysis is considered a self-limiting condition, with a favorable prognosis if diagnosed and treated promptly and accurately (3). Research indicates that approximately two-thirds of patients may experience abnormal regeneration of the facial nerve, often accompanied by synkinesis and crocodile tears. Poor recovery of facial muscle control in some patients can lead to facial deformity and pain (4).

Various treatment modalities are available for peripheral facial paralysis, including symptomatic treatment in Western medicine (5, 6), external treatment in traditional Chinese medicine (7–9), and combination therapies (10, 11). Electroacupuncture is a commonly used external treatment, supported by evidence from some review studies (8, 12). However, existing research often focuses on the influence of electroacupuncture parameters such as waveform, frequency, and intervention timing on the treatment of peripheral facial paralysis. TDP (Teding Diancibo Pu) therapy, also known as the “Magic Lamp” or “Infrared Therapeutic Apparatus” (13), is a domestically developed electromagnetic spectrum therapy device in China. TDP emits micron-level electromagnetic waves, exerting effects such as promoting blood circulation, relieving stasis, and alleviating pain. The elements generated by TDP radiation can enhance endogenous enzyme activity, promote metabolism, and boost immunity. Additionally, TDP irradiation can increase the content of endorphins in the body, alleviating pain (14).

In recent years, numerous studies have focused on the use of electroacupuncture combined with TDP therapy for the treatment of peripheral facial paralysis. However, the results of these studies have been inconsistent due to variations in sample sizes and differences in study design (15–17). Additionally, there is a lack of robust evidence regarding the comparative efficacy of electroacupuncture combined with TDP therapy versus standalone electroacupuncture, conventional Western medications, or acupuncture in the treatment of peripheral facial paralysis. Therefore, this study aims to conduct a systematic review and Meta-analysis of randomized controlled trials on the use of electroacupuncture combined with TDP therapy for the treatment of peripheral facial paralysis. This evaluation will provide evidence-based support for the clinical implementation of electroacupuncture combined with TDP therapy in treating peripheral facial paralysis.

## 2 Materials and methods

### 2.1 Literature search strategy

A systematic search was conducted in PubMed, EMBASE, Cochrane Library, CNKI, VIP, and Wanfang databases for studies on the use of electroacupuncture combined with TDP therapy for peripheral facial paralysis published up to April 29, 2024. The search terms in Chinese included “peripheral facial palsy,” “facial neuritis,” “Bell’s palsy,” “Bell’s facial paralysis,” “idiopathic facial neuritis”; “electroacupuncture”; “TDP,” “hot lamp,” “magic lamp,” “electromagnetic wave,” and “infrared.” The search terms in English included “Bell’s Palsy,” “peripheral facial palsy,” “facial paralysis,” “facial neuritis”; “electroacupuncture”; and “TDP.”

### 2.2 Inclusion and exclusion criteria

Inclusion criteria: (1) study type: randomized controlled trials published in Chinese or English; (2) subjects: patients diagnosed with peripheral facial paralysis with detailed diagnostic criteria; (3) interventions: electroacupuncture combined with TDP therapy; the control group receiving other treatments such as standalone electroacupuncture, conventional Western medications, or acupuncture; and (4) outcomes: clinical efficacy including overall response rate and cure rate, facial nerve function score, facial disability index score, and adverse effects.

Exclusion criteria: (1) studies that were duplicate reports or from which valid data could not be extracted; (2) case reports, reviews, and conference abstracts; and (3) animal or cadaver studies.

### 2.3 Information extraction and quality evaluation

Two independent investigators meticulously screened the literature and extracted data in strict accordance with the inclusion and exclusion criteria. In cases of disagreement, discussions were held to reach a consensus. The extracted data included the first author, year of publication, geographical background, sample size, intervention measures, and outcome indicators.

The modified Jadad scale (18) was employed to evaluate the quality of the literature. This scale assesses literature quality based on the randomization method, whether allocation concealment was present, the correct implementation of blinding, and the description of withdrawals and dropouts. Mention “random,” “random allocation” and “random grouping” and so on, and score 1 point; If the use of’ double-blind’ is mentioned as 1 point, the double-blind method is correctly described as 2 points; The reasons and cases of withdrawal and loss of follow-up in each group were reported, and the number of cases was 1 point. Studies scoring 4–7 points were considered high-quality research, those scoring 1–2 points were deemed low-quality, and studies scoring 0 points were excluded from the research. The quality evaluation was conducted independently by two researchers, and any discrepancies were resolved through discussion to determine the final score.

### 2.4 Statistical analysis

Meta-analysis was performed using Stata15.0 statistical software. For categorical data, the effect size was estimated using the risk ratio (RR) and its 95% confidence interval (CI). For continuous data, the standardized mean difference (SMD) and its 95% CI were utilized. The *I^2^* statistic was used to evaluate heterogeneity due to non-threshold effects. Specifically, when *I^2^* ≥ 50%, the DerSimonian and Laird random-effects model was employed for meta-analysis; when *I^2^* < 50%, the fixed-effects model was used.

## 3 Results

### 3.1 Literature screening results

**Figure 1:**
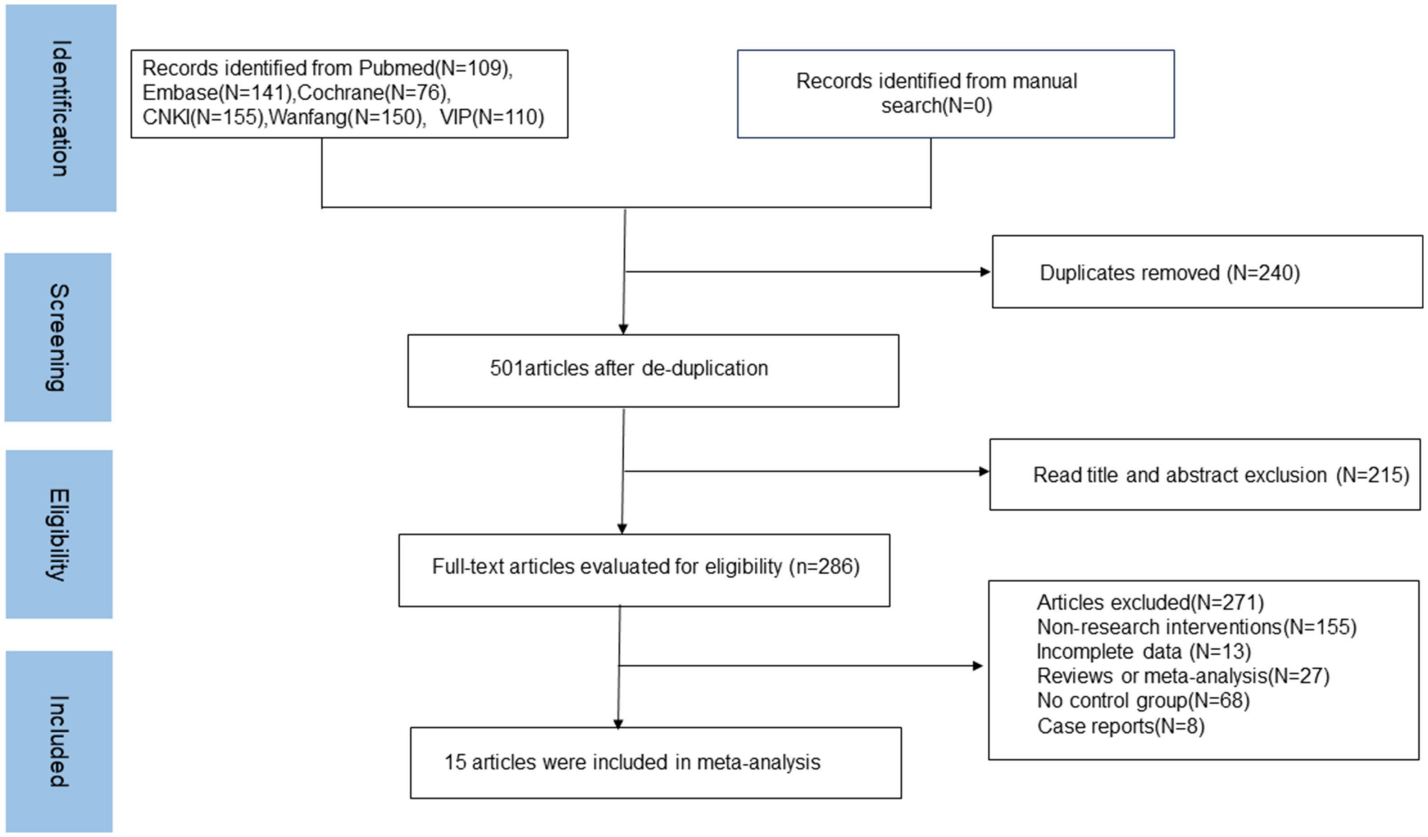
Literature screening flowchart and results.

A total of 741 articles were retrieved for this study. After removing 240 duplicate articles, 215 articles were excluded based on their titles and abstracts for being irrelevant. The remaining 286 articles were subjected to full-text screening, resulting in the inclusion of 15 articles in the meta-analysis. The literature screening process and results are depicted in Figure 1.

### 3.2 Basic information of included studies

A total of approximately 1,744 participants were included in all studies (1,008 in the treatment group and 736 in the control group). Table 1 summarizes the basic characteristics of the included studies. The study subjects were all from China. In 13 studies, the intervention for the treatment group was electroacupuncture combined with TDP (15–17, 19–27), while in two studies, the intervention was electroacupuncture point-through-point combined with TDP (28, 29). The control group interventions varied and included electroacupuncture (16, 19, 21–23, 25), acupuncture (15, 20), acupuncture combined with electroacupuncture (15, 20), Western medicine (17, 24), TDP therapy (26), electroacupuncture point-through-point (27), and acupuncture combined with TDP therapy (30). The majority of studies included participants who were minors (*N* = 11) (15–17, 21, 22, 24–27, 29, 30), while the remaining studies focused exclusively on adult subjects (*N* = 4) (19, 20, 23, 28).

**Table 1:** Basic characteristics of the 15 included studies.

Author	Year	Area	Treatment group			Control group			Outcome
			Sample size	Age	Intervention	Sample size	Age	Intervention	
Xiao Huizhong	1990	Fujian Province	36	–	Electroacupuncture + TDP	36	–	Electroacupuncture	①②
Che Juan	1993	Heilongjiang Province	180	20 ~ 40	Electroacupuncture + TDP	40	20 ~ 40	Acupuncture	①②
Zhu Qiufen	1994	Gansu Province	90	10 ~ 59	Electroacupuncture + TDP	80	10 ~ 59	TDP	①②
Wu Hongbo	1998	Fujian Province	132	21 ~ 68	Electroacupuncture + TDP	68	21 ~ 68	Electroacupuncture	①②
Qiu Xiaolong	2006	Zhejiang Province	38	15 ~ 80	Electroacupuncture + TDP	43	15 ~ 80	Acupuncture + TDP	①②
Zhang Guangli	2008	Hunan Province	50	13 ~ 72	Electroacupuncture point-through-point + TDP	50	15 ~ 69	Electroacupuncture point-through-point	①②
Tian Qiang	2009	Guizhou Province	18	1 ~ 50	Electroacupuncture + TDP	22	7 ~ 60	Electroacupuncture	①②
Xin Yuwen	2010	Guangdong Province	65	3 ~ 75	Electroacupuncture + TDP	30	5 ~ 76	Western medicine	①②
Chang Xueli	2011	Henan Province	50	27.26 ± 15.78	Electroacupuncture + TDP	22	26.87 ± 16.25	Acupuncture	①②
Cao Fadong	2012	Henan Province	85	19 ~ 68	Electroacupuncture + TDP	85	20 ~ 69	Electroacupuncture	①②
Hu Jiaqian	2013	Zhejiang Province	48	3 ~ 70	Electroacupuncture + TDP	44	14 ~ 73	Electroacupuncture	①②
Wu Lei	2013	Chongqing	88	3 ~ 7	Electroacupuncture + TDP	88	3 ~ 7	Acupuncture + TDP	①②
Zou Yan	2014	Zhejiang Province	30	15 ~ 55	Electroacupuncture + TDP	30	18 ~ 59	Electroacupuncture + acupuncture	①②
Li Shujuan	2015	Hebei Province	76	16 ~ 72	Electroacupuncture + TDP	76	14 ~ 69	Electroacupuncture	①②
Ge Xiaohang	2016	Henan Province	22	22 ~ 78	Electroacupuncture point-through-point + TDP	22	18 ~ 71	Electroacupuncture point-through-point	①②

TDP, Teding Dianci Pu; ①: Efficacy rate, ②: Cure rate.

### 3.3 Assessment of the quality of included studies

To evaluate the quality of the studies included, we utilized the Jadad scale. Detailed findings are presented in Table 2. The specific scores of the 15 included studies are as follows: there were 2 studies rated as high-quality literature with scores ranging from 4 to 7, both scoring 4 points. Thirteen studies were classified as low-quality literature with scores ≤3, including 2 studies scoring 3 points, 8 studies scoring 2 points, and 3 studies scoring 1 point. Generation of random sequences: Two studies employed randomization and described the correct randomization methods, while eight studies used randomization but did not describe the methods. Randomization concealment: Two studies only mentioned using random number methods or random number tables for random allocation but did not indicate whether this method prevented clinicians and participants from predicting the allocation sequence. Usage of blinding: None of the studies mentioned whether blinding was used.

**Table 2:** Quality assessment of included studies.

Author	Year	Random	Randomization concealment	Blind	Withdrawal/loss to follow up	Total score
Xiao Huizhong	1990	0	0	0	1	1
Che Juan	1993	1	0	0	1	2
Zhu Qiufen	1994	1	0	0	1	2
Wu Hongbo	1998	1	0	0	1	2
Qiu Xiaolong	2006	1	0	0	1	2
Zhang Guangli	2008	1	0	0	1	2
Tian Qiang	2009	2	1	0	1	4
Xin Yuwen	2010	1	0	0	1	3
Chang Xueli	2011	0	0	0	1	1
Cao Fadong	2012	2	0	0	1	3
Hu Jiaqian	2013	0	0	0	1	1
Wu Lei	2013	2	0	0	1	3
Zou Yan	2014	1	0	0	1	2
Li Shujuan	2015	1	0	0	1	2
Ge Xiaohang	2016	2	1	0	1	4

### 3.4 Meta-analysis results

#### 3.4.1 Efficiency rate

**Figure 2:**
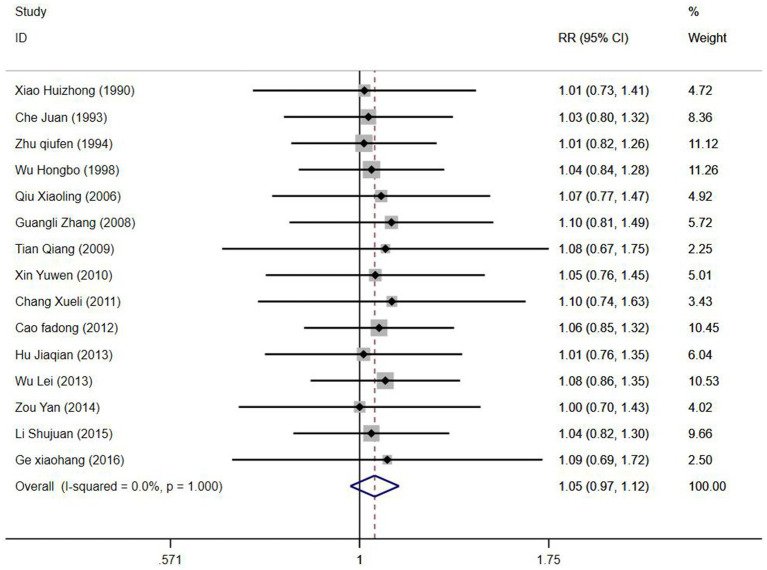
Systematic review and meta-analysis of efficacy rate.

**Figure 3:**
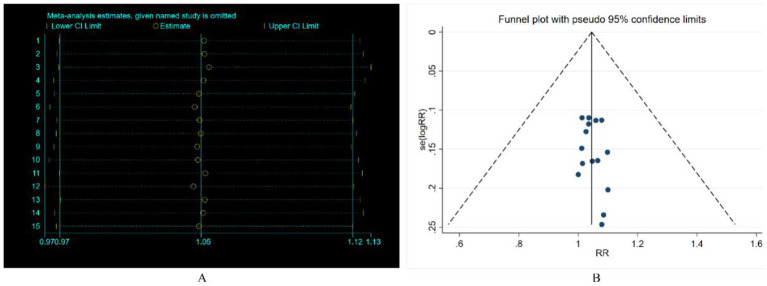
Sensitivity analysis and funnel plot of efficacy rate.

**Figure 4:**
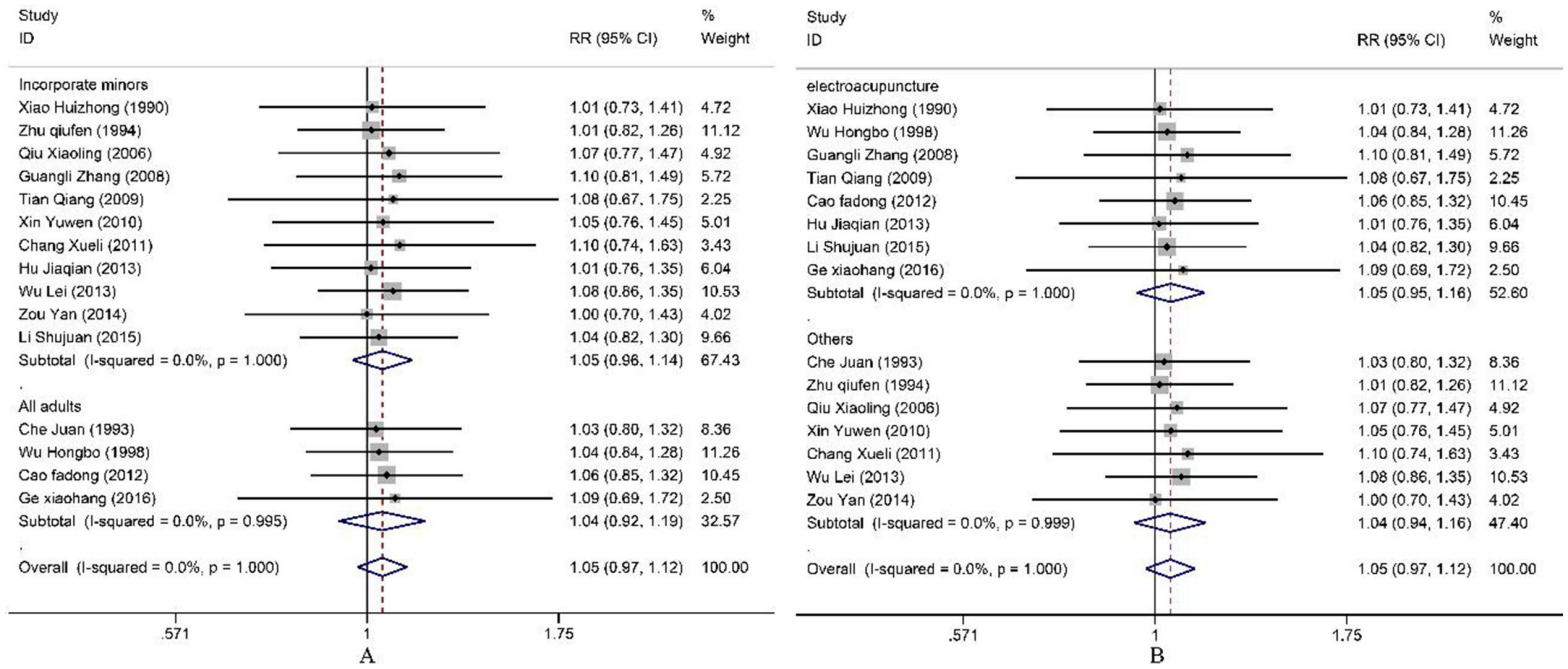
Subgroup analysis.

**Figure 5:**
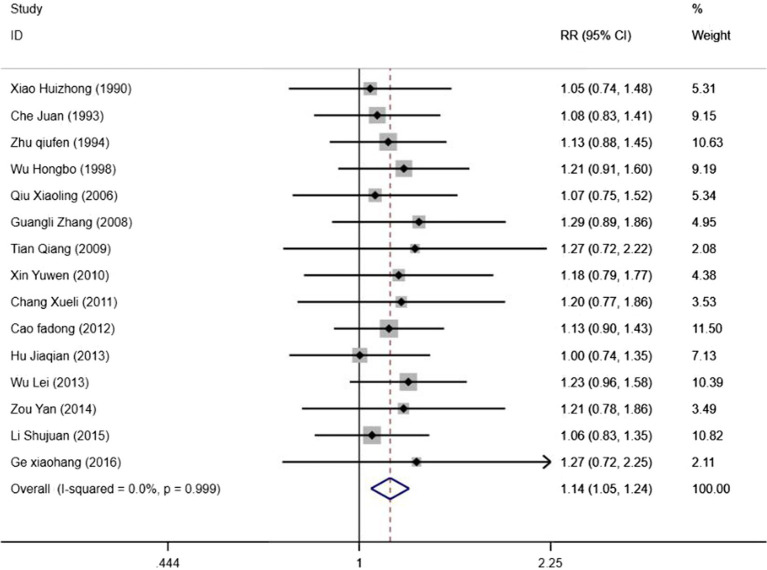
Systematic overview and meta-analysis of cure rate.

All included studies reported the overall efficacy rate. Heterogeneity test analysis indicated no statistical heterogeneity among the 15 studies (*p* = 1.00, *I^2^* = 0%), thus employing a fixed-effects model for pooled analysis. Meta-analysis results (Figure 2) revealed no significant difference in overall efficacy rate between electroacupuncture combined with TDP treatment for peripheral facial paralysis and other treatment modalities [*OR* = 1.05, 95%*CI* (0.97, 1.12), *p* = 0.226]. Sensitivity analysis was conducted to assess the stability of the study findings. The results (Figure 3) indicated no significant changes, suggesting stability. Additionally, Egger’s test (*T* = 0.98, *p* = 0.345) and funnel plot results revealed no apparent publication bias (Figure 4). Stratification based on factors such as the inclusion of minors and whether electroacupuncture was used as a control intervention showed no significant variations in study outcomes (Figure 5).

#### 3.4.2 Cure rate

**Figure 6:**
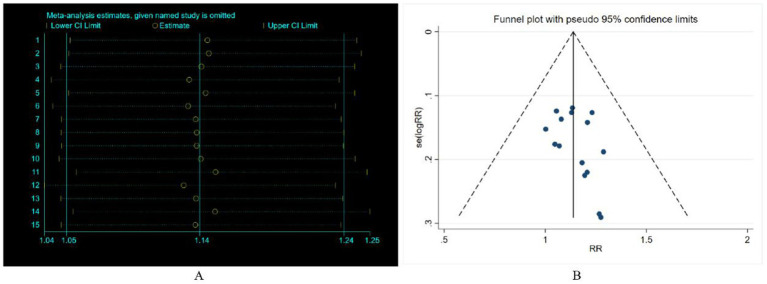
Sensitivity analysis and funnel plot of cure rate.

**Figure 7:**
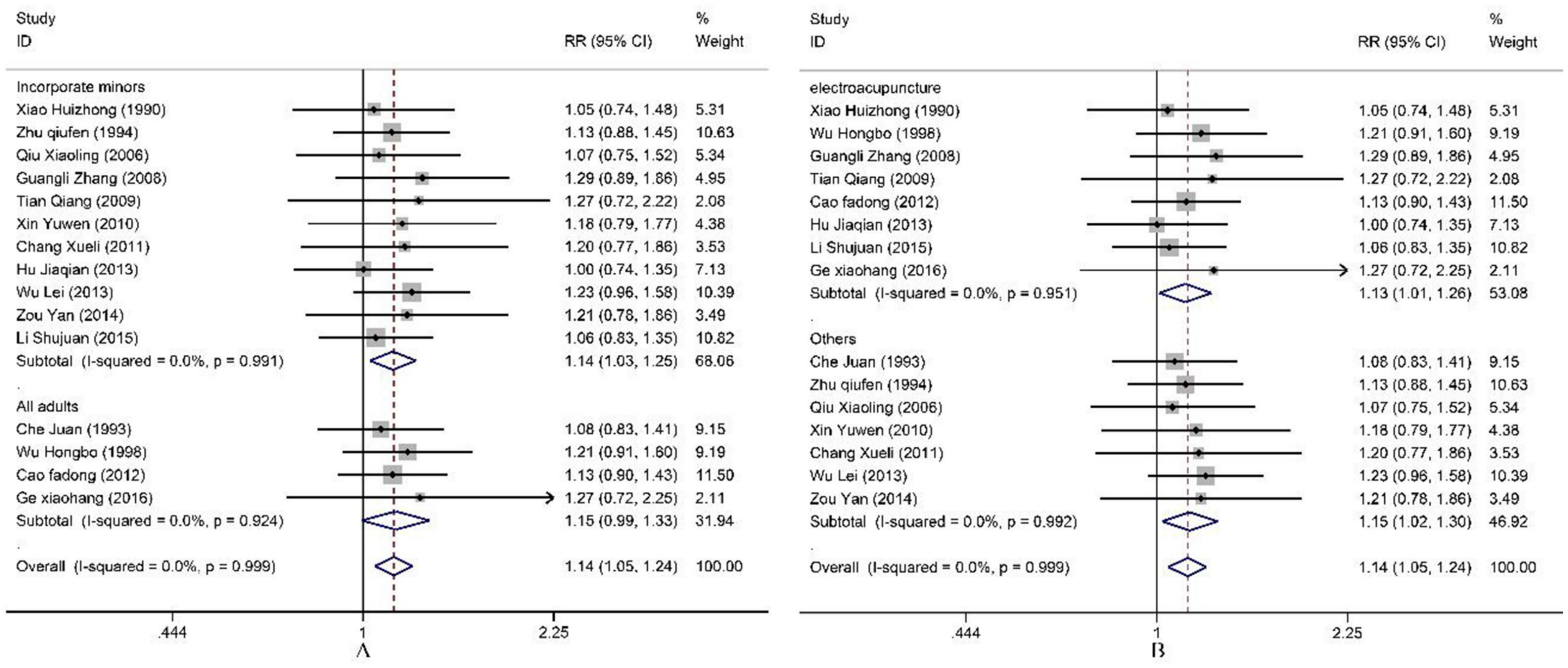
Subgroup analysis of cure rate.

All included studies reported both cure rate and efficacy rate. Consequently, our study pooled the results for the cure rate. Heterogeneity testing revealed no statistical heterogeneity among the 15 studies (*p* = 1.00, *I^2^* = 0.966%). Therefore, a fixed-effect model was employed for the combined analysis. The Meta-analysis results (Figure 5) demonstrate that electroacupuncture combined with TDP therapy significantly outperformed other treatment methods in terms of cure rate [*OR* = 1.14, 95%*CI* (1.05, 1.24), *p* = 0.002]. To assess the robustness of these findings, a sensitivity analysis was conducted (Figure 6). The results indicate that there were no significant changes, suggesting that the results are stable. Additionally, Egger’s test (*T* = 1.42, *p* = 0.180) and the funnel plot results revealed no significant publication bias (Figure 6). When stratified by the inclusion of minors in the study population, it was observed that in studies including minors, the combination of electroacupuncture and TDP therapy demonstrated superior efficacy in treating peripheral facial paralysis compared to other therapeutic modalities [*OR* = 1.14, 95% *CI* (1.03, 1.25), *p* = 0.011]. Conversely, in studies where the population comprised solely adults, no significant difference was found between the combination therapy and other treatments [*OR* = 1.15, 95% *CI* (0.99, 1.33), *p* = 0.059] (Figure 7). Stratification based on whether the control group interventions included electroacupuncture did not alter the results significantly. Regarding the rate of marked improvement, electroacupuncture combined with TDP therapy was superior to both monotherapy [*OR* = 1.13, 95% *CI* (1.01, 1.26), *p* = 0.032] and other therapeutic methods [*OR* = 1.15, 95% *CI* (1.02, 1.30), *p* = 0.020].

## 4 Discussion

Peripheral facial paralysis is an inflammatory disorder induced by various etiologies that result in facial nerve impairment. Presently, the incidence rate of peripheral facial paralysis in China ranks sixth among all neurological disorders, with approximately 3 million cases annually (31), surpassing the annual incidence rate of 20 per 100,000 in Western countries (32).

In contemporary clinical practice, the predominant therapeutic approach for patients with peripheral facial paralysis involves pharmacological treatment. However, medication cycles typically require a longer recovery time, with poorer outcomes and prognosis for quality of life. In comparison, external treatments in traditional Chinese medicine exhibit significant therapeutic advantages in the clinical treatment of peripheral facial paralysis (33–35). Electroacupuncture demonstrates considerable benefits over conventional Western medicine (36) and acupuncture alone (30). Furthermore, the thermal effects generated by TDP electromagnetic waves have biological effects such as enhancing metabolism, improving microcirculation in lesioned tissues, reducing inflammation, providing analgesic effects, and boosting immunity (37). TDP has been proven to have significant therapeutic effects in many clinical fields, with its application becoming increasingly widespread. Therefore, this study aims to conduct a systematic review and meta-analysis of randomized controlled trials on electroacupuncture combined with TDP for the treatment of peripheral facial paralysis, to evaluate the effectiveness of electroacupuncture combined with TDP in treating peripheral facial paralysis.

The findings of this study indicate that the efficacy rate of electroacupuncture combined with TDP therapy for treating peripheral facial paralysis shows no significant difference when compared to other therapeutic methods. This consistency in results persists across various subgroups. There is a certain heterogeneity in this study, which is mainly caused by the different measures of the control group, the inconsistent evaluation criteria of curative effect and the small sample size. Notably, the recovery rate for peripheral facial paralysis treated with electroacupuncture combined with TDP therapy surpasses that of other methods. Subgroup analysis further reveals that, among pediatric populations, the recovery rate for electroacupuncture combined with TDP therapy is superior to other treatments. Regardless of whether the comparison is with standalone electroacupuncture or alternative therapies, the recovery rate for electroacupuncture combined with TDP therapy remains higher. However, it is crucial to note that the methodological quality and reporting standards of the included studies are generally low, and the sample sizes are limited, which constrains the overall reliability of this systematic review. In this sense, large randomized controlled trials with higher quality requirements need to be conducted to validate the efficacy of electroacupuncture combined with TDP in the treatment of peripheral facial palsy.

There are several limitations inherent in this study. Firstly, both the treatment and control groups utilized electroacupuncture for peripheral facial paralysis; however, there was no description of the waveform or timing of the electroacupuncture application. Variations in waveform (38, 39) and timing (40, 41) can significantly impact the efficacy of treatment for peripheral facial paralysis. Secondly, the control group was subjected to multiple therapeutic interventions, which may have confounded the results and affected their generalizability. The sample size of the included research is small, and the research results may lack representativeness. Furthermore, none of the included studies employed blinding techniques, potentially introducing a degree of bias into the findings. It is suggested that large-scale, high-quality randomized controlled trials should be carried out in future research, and stricter blind method and randomization procedures should be included. In addition, it is suggested that the measurement of the results should be further standardized.

## 5 Conclusion

Meta-analysis showed that the effective rate of electroacupuncture combined with TDP in the treatment of peripheral facial paralysis was not significantly different from other treatment methods, and the recovery rate was better than other treatment methods. Subgroup analysis showed that the recovery rate of electroacupuncture combined with TDP in the treatment of peripheral facial paralysis was better than other methods in the minor population; whether electroacupuncture alone or other treatment methods, the recovery rate of electroacupuncture combined with TDP in the treatment of peripheral facial paralysis was better than other methods.

## Data Availability

The original contributions presented in the study are included in the article/Supplementary material, further inquiries can be directed to the corresponding author.

## References

[1] Baugh RF Basura GJ Ishii LE Schwartz SR Drumheller CM Burkholder R . Clinical practice guideline: Bell's palsy. Otolaryngol Head Neck Surg. (2013) 149:S1–S27. doi: 10.1177/0194599813505967 24189771

[2] Yunyun B Lin C Yiwu D Chen L Zhang ZQ Bu YY . Clinical guidelines for the treatment of idiopathic facial paralysis in China (2022 edition). Neural Injury Funct Reconstruct. (2023) 18:1–12. doi: 10.16780/j.cnki.sjssgncj.20220639

[3] de Almeida JR Guyatt GH Sud S Dorion J Hill MD Kolber MR . Management of Bell palsy: clinical practice guideline. CMAJ. (2014) 186:917–22. doi: 10.1503/cmaj.131801, PMID: 24934895 PMC4150706

[4] Reich SG. Bell’s palsy. Continuum. (2017) 23:447–66. doi: 10.1212/CON.0000000000000447 28375913

[5] Han PJ Guo KF He JY Jin GX. Observation on the efficacy of facial muscle functional training combined with hormone treatment for acute stage Bell's facial paralysis. J Med Theory Pract. (2021) 34:2773–5. doi: 10.19381/j.issn.1001-7585.2021.16.012

[6] Zhang HY. Effect of small needle knife cooperated with hyperbaric oxygen and neurotrophic drugs in the treatment of intractable facial paralysis. China Med Herald. (2017) 14:163–6.

[7] Fang LJ Xu Y Wang CY. Research Progress on acupuncture and Moxibustion for sequelae of peripheral facial paralysis. New Chin Med. (2023) 55:190–3. doi: 10.13457/j.cnki.jncm.2023.03.042

[8] Wang WH Jiang RW Liu NC. Electroacupuncture is effective for peripheral facial paralysis: a meta-analysis. Evid Based Complement Alternat Med. (2020) 2020:5419407. doi: 10.1155/2020/5419407 32328134 PMC7150689

[9] Yang LS Zhou DF Zheng SZ Zhao BM Li HG Chen QQ . Early intervention with acupuncture improves the outcome of patients with Bell's palsy: a propensity score-matching analysis. Front Neurol. (2022) 13:943453. doi: 10.3389/fneur.2022.943453 36188388 PMC9517937

[10] Chen WQ Li Q. Electroacupuncture combined with Qianzhengsan decoction for the treatment of peripheral facial paralysis: a retrospective study. Medicine. (2022) 101:e30740. doi: 10.1097/MD.0000000000030740, PMID: 36123862 PMC9478275

[11] Liu X. Clinical observation on 60 cases of facial neuritis treated by Electroacupuncture combined with microwave and intermediate frequency pulse electric. China Med Device Informat. (2022) 28:131–3. doi: 10.3969/j.issn.1006-6586.2022.02.044

[12] Yu J Sun ZR Li HL. Consideration on intervening time of acupuncture and Moxibustion in treating peripheral facial paralysis as well as diversification of treatment methods. J Shandong Univ Tradit Chin Med. (2020) 44:333–8. doi: 10.16294/j.cnki.1007-659x.2020.03.022

[13] Zhao HT. Failure analysis and maintenance of TDP. Med Equip. (2015) 7:125. doi: 10.3969/j.issn.1002-2376.2015.07.078

[14] Barolet D Christiaens F Hamblin MR. Infrared and skin: friend or foe. J Photochem Photobiol B. (2016) 155:78–85. doi: 10.1016/j.jphotobiol.2015.12.014, PMID: 26745730 PMC4745411

[15] Chang XL. The observation of treating peripheral facial paralysis by electro-acupuncture plus TDP. Clin J Chin Med. (2011) 3:65–6. doi: 10.3969/j.issn.1674-7860.2011.13.035

[16] Li SJ Liu WH. Clinical analysis of 76 cases of peripheral facial paralysis treated with electroacupuncture plus microwave synthesis. J Hebei North Univ. (2015) 3:81–2. doi: 10.3969/j.issn.1673-1492.2015.03.022

[17] Zou Y Ma ZR Shen JP Liu WZ. Acupuncture combined with electroacupuncture therapy plus TDP irradiation for the treatment of peripheral facial paralysis in 30 cases. Tradit Chin Med Res. (2014) 27:62–3. doi: 10.3969/j.issn.1001-6910.2014.02.31

[18] Oremus M Wolfson C Perrault A Demers L Momoli F Moride Y. Interrater reliability of the modified Jadad quality scale for systematic reviews of Alzheimer's disease drug trials. Dement Geriatr Cogn Disord. (2001) 12:232–6. doi: 10.1159/000051263, PMID: 11244218

[19] Cao FD. Clinical observation of electroacupuncture combined with TDP in the treatment of peripheral facial paralysis. Chin Commun Doct. (2012) 14:185. doi: 10.3969/j.issn.1007-614x.2012.23.170

[20] Che J Li L Zhao LZ. Effect of electroacupuncture and TDP irradiation in the treatment of 180 cases of peripheral facial paralysis. J Mudanjiang Med Coll. (1993) 1:32.

[21] Hu JQ Li KP. Electro-acupuncture plus TDP treating 48 cases of peripheral facial paralysis. Liaoning J Tradit Chin Med. (2013) 40:2574–5. doi: 10.13192/j.issn.1000-1719.2013.12.074

[22] Tian Q Li Y Zhang SZ Luo CL Wang YJ Nie ZH. Analysis of the therapeutic effect of electroacupuncture with TDP lamp irradiation in the treatment of peripheral facial paralysis. J Zunyi Med Univ. (2009) 32:467–70. doi: 10.3969/j.issn.1000-2715.2009.05.012

[23] Wu HB. Therapeutic effect of electroacupuncture with TDP in the treatment of peripheral facial paralysis in 132 cases. Fujian Med J. (1998) 20:96-96.

[24] Wu L. Observations on 88 cases of acute peripheral facial paralysis in young children treated with electroacupuncture. Proceedings of the 4th International Science and Technology Conference on the Modernization of Traditional Chinese Medicine. (2013):1–5.

[25] Xiao HZ. Comparison of the efficacy of electroacupuncture and TDP in the treatment of peripheral facial paralysis in 72 cases. J Sichuan Tradit Chin Med. (1990) 3:50.

[26] Xin YW. Electroacupuncture and TDP treatment for facial nerve palsy in 65 cases. Inner Mong J Tradit Chin Med. (2010) 29:31–2. doi: 10.3969/j.issn.1006-0979.2010.10.040

[27] Zhu QF. Treatment of 90 cases of acute facial neuritis with TDP plus electroacupuncture. Chin Acupunct Moxibust. (1994) S1:421.

[28] Ge XH Meng FD Liu LX. Observation on the curative effect of penetration needling combined with TDP in the treatment of 22 patients with peripheral facial nerve paralysis. Chine Commun Doct. (2016) 32:87–8. doi: 10.3969/j.issn.1007-614x.2016.1.54

[29] Zhang GL. Effect of electroacupuncture point-through-point and TDP in the treatment of peripheral facial neuritis in 50 cases. Chin J Tradit Med Sci Technol. (2008) 15:4. doi: 10.3969/j.issn.1005-7072.2008.02.070

[30] Qiu XL. Effect of electroacupuncture with TDP lamp in the treatment of facial nerve paralysis in 78 cases In: The fifth academic seminar of neurology professional Committee of Zhejiang Association of integrated Chinese and Western medicine integrated neurology class. Ninghai, Zhejiang, China: (2006). 2.

[31] Tm O. Medical Management of Acute Facial Paralysis. Otolaryngol Clin N Am. (2018) 51:1051–75. doi: 10.1016/j.otc.2018.07.004 30297178

[32] Neurology Branch of the Chinese Medical Association, neuromuscular disease Group of Neurology Branch of the Chinese Medical Association, Electromyography and Clinical Neurophysiology Group of Neurology Branch of the Chinese Medical Association . Chinese guidelines for the diagnosis and treatment of idiopathic facial nerve palsy. Chin J Neurol. (2016) 2:84–6. doi: 10.3760/cma.j.issn.1006-7876.2016.02.002

[33] Li M Zhu SS Wan QR Ruan JG Wang YJ Xu TS . Effect of warm acupuncture at Yifeng (TE 17) on facial paralysis with periauricular pain during pregnancy. Zhongguo Zhen Jiu. (2020) 40:1281–5. doi: 10.13703/j.0255-2930.20191128-k0002, PMID: 33415868

[34] Wang B Wang JW Li H. Observation on therapeutic effect of peripheral facial paralysis treated by string puncture combined with electroacupuncture. Zhongguo Zhen Jiu. (2011) 31:47–50. PMID: 21355158

[35] Zhu P Wang H Zhang L Jiang X. Deep learning-based surface nerve electromyography data of E-health Electroacupuncture in treatment of peripheral facial paralysis. Comput Math Methods Med. (2022) 2022:8436741–12. doi: 10.1155/2022/8436741, PMID: 35685899 PMC9173966

[36] Xiao SH. Clinical effects of electroacupuncture in the treatment of peripheral facial paralysis. Bao Jian Wen Hui. (2023) 24:61–4.

[37] Ye C. Clinical observation on curative of massage combined with TDP in the treatment of tibial fatigue periostitis. Guangdong: Guangzhou University of Traditional Chinese Medicine (2017).

[38] Tong ZZ. Clinical observation of low-frequency electrical stimulation in the treatment of facial paralysis. Master. (2012)

[39] Chen Q Chen F Li YN Bao WT Zhang H. Clinical efficacy of electroacupuncture dilatational wave treatment of acute-stage facial paralysis. Acupunct Electrother Res. (2021) 46:405–18. doi: 10.3727/036012921X16237619666085

[40] Qu QW Xiong T. Clinical observation on electroacupuncture for treatment of peripheral facial paralysis at different stages. Zhongguo Zhen Jiu. (2005) 25:323–5. PMID: 16320747

[41] Xiao X Zheng Q Shi Y Zhang L Zhao L Zhou S . Association of Patients’ characteristics with acupuncture treatment outcomes in treating Bell's palsy: results from a randomised controlled trial. Evid Based Complement Alternat Med. (2019) 2019:1–7. doi: 10.1155/2019/6073484, PMID: 31511780 PMC6714330

